# Quantitative geospatial dataset on the near-surface heavy metal concentrations in semi-arid soils from Maibele Airstrip North, Central Botswana

**DOI:** 10.1016/j.dib.2016.08.026

**Published:** 2016-08-17

**Authors:** Peter N. Eze, Valiant S. Mosokomani, Theophilus K. Udeigwe, Opeoluwa F. Oyedele

**Affiliations:** a Department of Earth & Environmental Science Botswana International University of Science & Technology, Private Bag 016, Palapye, Botswana; b Independent scholar, 8901 Avenue T, Lubbock, TX 79423, USA; c Department of Mathematics & Statistics, Namibia University of Science & Technology, Windhoek, Namibia

**Keywords:** Heavy metals, Cu–Ni exploration, Grid sampling, Meta sedimentary, Semi-arid soils

## Abstract

This article contains a statistically analyzed dataset of the heavy metals including Cr, Co, Ni, Cu, Zn and Pb contents of near-surface (~30 cm depth) soils in a Cu–Ni prospecting field at Airstrip North, Central Botswana. The soils developed on paragneisses and amphibolites parent materials in a semi-arid environment with hardveld vegetation, “The geology of the Topisi area” (Key et al., 1994) [1]. Grid sampling was adopted in the field data collection. Heavy metals were determined using the relatively new portable x-ray fluorescence spectrometer (Delta Premium, 510,890, USA) technology in a “soil” mode. The data presented was obtained from the average reading of two soil samples collected from same point but passed through sieves.

**Specifications Table**

**Table:** 

Subject area	*Environmental Science*
More specific subject area	*Soil geochemistry*
Type of data	*Table, figure*
How data was acquired	*Survey, portable x-ray fluorescence (Delta Premium, Rh Tube Anode Instrument Serial Number: 510,890, USA); Fieldmaster® soil sampling sieve set*
Data format	*Raw, analyzed*
Experimental factors	*Soil samples were collected at a 30 cm depth to avoid contamination by surficial anthropogenic deposits and organic matter.*
Experimental features	*Determine the concentration levels of heavy metals including Cr, Co, Ni, Cu, Zn and Pb.*
Data source location	*Maibele Airstrip North, Central Botswana.*
Data accessibility	*Data is with this article*

**Value of the data**

• It can serve as a geochemical base-line data for heavy metals concentrations in near-surface soils developed on meta sedimentary parent materials in semi-arid environments.
• As a dataset, it could be used for pedogenic and geostatistical modeling and simulation of heavy metals concentration in semi-arid soils.
• Having been geo-referenced, data can be used for GIS modeling and environmental impact assessment studies by environmental scientists.
• Useful as a good lead in mineral prospecting and geochemical exploration by geologists.

## 1 Data

**Fig. 1:**
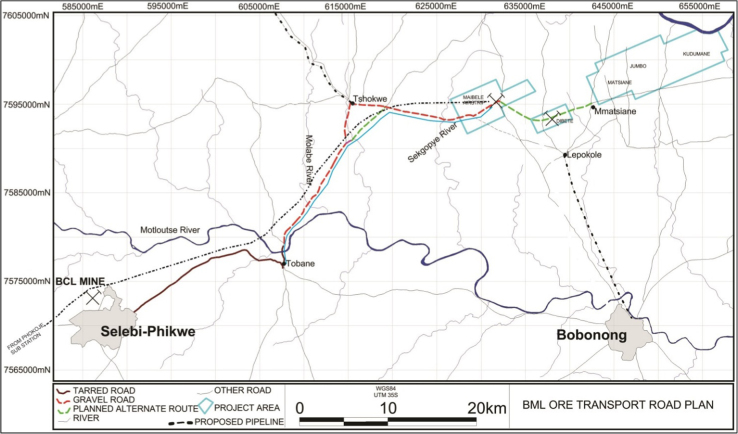
A map showing the geographical location of the studied area (Maibele).

Herein, the data consists of tables and figures which help analyze the near-surface (~30 cm depth) heavy metals contents of soils collected from 1050 geo-referenced points underlain by paragneisses and amphibolites parent materials [1] at the Maibele Airstrip North in Central Botswana (Fig. 1). Other heavy metals below detection limit (dl) including Mo, dl <5 ppm; Cd, dl <10 ppm; Sn, dl <20 ppm; Sb, dl <20 ppm; W, dl <10 ppm; U, dl <5 ppm; and Se, dl <5 ppm had no values reported in the data (Supplementary Table 1). Portable x-ray fluorescence spectrophotometer in a “soil” mode was used to determine the heavy metals. The average of two readings on two samples (sieved and non-sieved) collected from the same point on the grid layout was recorded and reported.

## 2 Experimental design, materials and methods

**Fig. 2:**
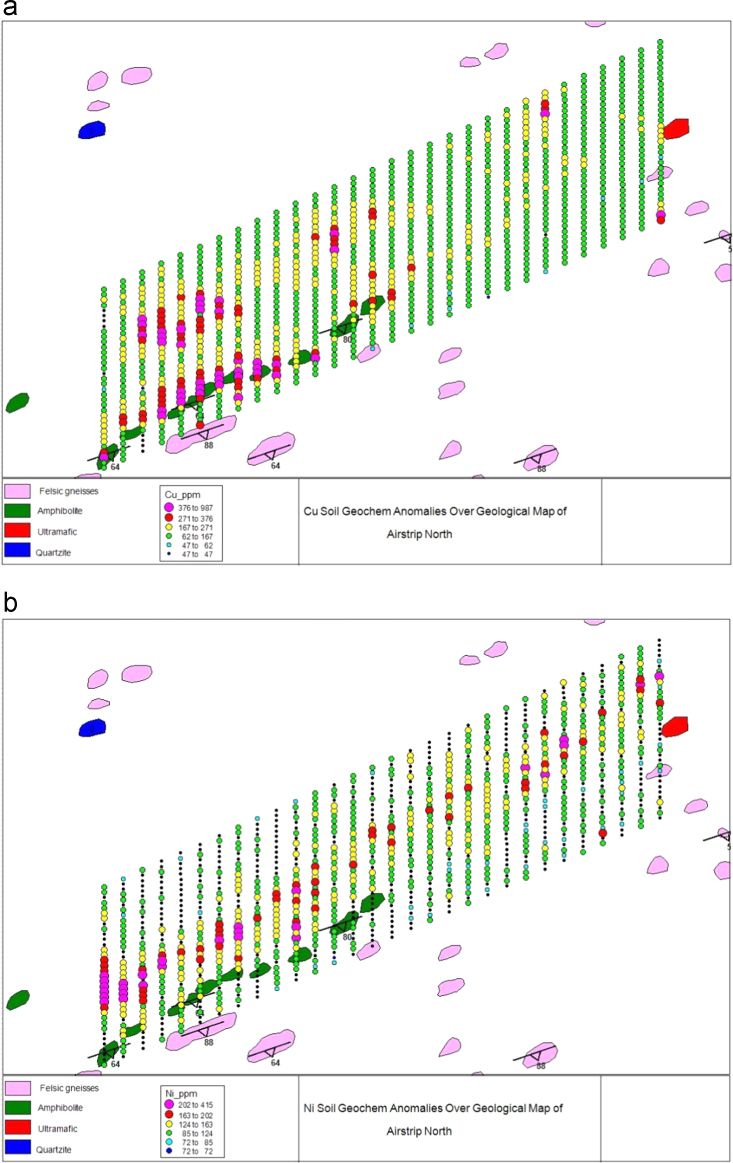
Elemental anomalies orientation (trend) of all the 30 sample lines and the 35 samples points for each line. (a) Cu and (b) Ni.

Soil samples were collected at intervals of 25 m (thus a sample spacing of 25 m) following straight marked lines. Sample line trend was from north to south and a total of 30 lines were sampled, each with 35 sampling points (Fig. 2a and b).

A total distance of about 875 m was covered for each line. A pit of about 30 cm depth was dug to remove the topsoil (Ap horizon) and organic material before collecting soil samples. Two soil samples were collected for each point, one sample was sieved using the Fieldmaster soil sampling sieve set before being placed in a labeled transparent sample bag and the other was put in a sample bag as collected (not sieved). The two samples collected at a single point were label with the same sample number but differentiate by letters at the end (for example, 1105451a and 1105451b).

All soil samples were taken to the base camp and allowed to air dry before analyzing using a portable x-ray fluorescence analyzer. Samples from the same point were analyzed consecutively, and the analyzer made an average analysis from the measurements it obtained from the two samples. The data was downloaded into a computer and an excel document showing the element contents for each sample was made (Supplementary Table 1).

A calibration standard (for this analysis AMIS0329 and AMIS0316), a blank and a duplicate were used after every 20 samples were analyzed, and they are highlighted with a yellow color in the excel data sheet in Supplementary table.

### 2.1 Correlation analysis on the data

The correlation values obtained from Karl Pearson׳s correlation analysis of the data are given in Table 1 below. This table shows the estimated strength of the relationships between the six heavy metals. A correlation value that is closer to (±) 1 can be said to have stronger relationship strength, where the (±) indicates the direction of the relationship – with “+” denoting positive and “−” denoting negative. A “0” value indicates that there is no correlation between the respective variables.

### 2.2 Principal component analysis of the data

#### 2.2.1 Number of factors and identifying the variables under each factor

The loading values obtained from the PCA of the data are given in Table 2. This table shows the amount of variation that a particular variable contributed to a given factor. A variation value that is more than (±) 0.5 can be said to be a significant contribution to the respective factor. The contribution may vary from moderate to very high, with “0.6 and above” denoting high to very high contribution and “0.5–0.6” denoting moderate contribution.

From this table, if Kaiser׳s rule of taking the number of factors to use as the total number of eigenvalues ≥1 is applied, it can be deduced that the six heavy metals of the data can be grouped only under three factors.

From Table 2, heavy metals Co, Cu and Zn can be said to have contributed significantly to Factor 1, although with a moderate contribution of 0.521, 0.500 and 0.518 respectively. Likewise, only metals Cr and Ni contributed significantly to Factor 2, while only metal Pb contributed significantly to Factor 3. Thus, the six heavy metals of the data are grouped as follows:

First factor: {**Co**, **Cu**, **Zn**}
Second factor: {**Cr**, **Ni**}
Third factor: {**Pb**}.

Overall, the three factors explain 83.2% of the total variation in the data.

## Figures and Tables

**Table 1:** Correlation values of the data.

***n*****=1050**	**Cr**	**Co**	**Ni**	**Cu**	**Zn**	**Pb**
**Cr**	1					
**Co**	0.469	1				
**Ni**	0.528	−0.026	1			
**Cu**	0.163	0.430	−0.041	1		
**Zn**	0.310	0.733	−0.041	0.488	1	
**Pb**	0.102	0.198	0.028	0.520	0.274	1

**Table 2:** PCA loading values of the data.

**Heavy metals**	**Factor 1**	**Factor 2**	**Factor 3**
**Cr**	0.363	**−0.584**	0.061
**Co**	**0.521**	0.014	0.429
**Ni**	0.095	**−0.714**	−0.337
**Cu**	**0.500**	0.279	−0.321
**Zn**	**0.518**	0.130	0.324
**Pb**	0.336	0.233	**−0.700**
**Eigenvalues**	2.554	1.447	0.996
**Percentage of variance**	42.5%	24.1%	16.6%
**Cumulative percentage**	42.5%	66.7%	83.2%
